# Use of Transdermal Alcohol Sensors in Conjunction With Contingency Management to Reduce Alcohol Consumption in People With Alcohol Dependence Attending Alcohol Treatment Services: Protocol for a Pilot Feasibility Randomized Controlled Trial

**DOI:** 10.2196/57653

**Published:** 2024-07-31

**Authors:** Eileen Brobbin, Paolo Deluca, Stephen Parkin, Colin Drummond

**Affiliations:** 1 Department of Addiction Institute of Psychiatry, Psychology and Neuroscience King's College London London United Kingdom; 2 Department of Public Health Environments and Society at London School of Hygiene and Tropical Medicine King's College London London United Kingdom

**Keywords:** accuracy, addiction, alcohol, alcohol monitoring, alcohol treatment, contingency management, transdermal alcohol sensors, wearables, mobile phone, transdermal, TAS, wearable technology, alcohol use disorders, AUD, RCT, randomized controlled trial, abstinence, community-based, residential rehabilitation, consumption, alcohol consumption, low-risk consumption

## Abstract

**Background:**

Wearable technology for objective, continuous, and reliable alcohol monitoring has been developed. These are known as transdermal alcohol sensors (TASs). They can be worn on the wrist or ankle with the sensor pressed against the skin and can measure sweat vapors being emitted from the skin, to record transdermal alcohol concentration (TAC). Previous studies have investigated the accuracy and acceptability of the available TAS brands, but there has been little research into their use in people with alcohol use disorders (AUD).

**Objective:**

This feasibility randomized controlled trial aims to explore the feasibility, strengths, and limitations of using a TAS to monitor alcohol consumption in individuals in treatment for AUD with or without contingency management (CM) to promote abstinence or low-level alcohol consumption.

**Methods:**

The target sample size is 30 (15 randomized to each group). Participants will be recruited through poster adverts at alcohol services. Both groups (control and CM) will wear the TAS (BACtrack Skyn) for 2 weeks in the context of their usual treatment, meeting with the researcher every other weekday. In the last meeting, the participants will complete a postwear survey on their experience of wearing the TAS. The CM group will also receive small financial incentives for low or no alcohol consumption, as measured by the TAS. On days where the TAC peak is below a set threshold (<115.660 g/L), CM group participants will be rewarded with a £5 (US $6.38) voucher. There are financial bonuses if this target is achieved on consecutive days. The researcher will monitor TAC for each day of the study at each research visit and allocate financial incentives to participants according to a set reinforcement schedule.

**Results:**

The first participant was enrolled in June 2023, and the last in December 2023. Data analysis is underway and is estimated to be completed by June 2024. A total of 32 participants were enrolled.

**Conclusions:**

Most TAS brands have had limited application in clinical settings, and most studies have included healthy adults rather than people with AUD. TAS has the potential to enhance treatment outcomes in clinical alcohol treatment. The accuracy, acceptability, and feasibility of TAS for people with AUD in clinical settings need to be investigated. This is the first study to use TAS in specialized alcohol services with diagnosed AUD individuals currently receiving treatment from a south London alcohol service.

**Trial Registration:**

ISRCTN Registry ISRCTN46845361; https://www.isrctn.com/ISRCTN46845361

**International Registered Report Identifier (IRRID):**

DERR1-10.2196/57653

## Introduction

### Background

Many individuals require specialist alcohol treatment for their alcohol dependence, with the main aim being abstinence, although some may be able to aim for a goal of moderate or low-level alcohol consumption [1,2]. Specialist community alcohol services are free services provided by the National Health Service (NHS), delivered in the community (ie, not residential), offering a range of drug and alcohol treatments. The NHS is the publicly funded health care system in the United Kingdom. Following service engagement and assessment, the first step of specialist alcohol treatment will typically be medically assisted alcohol withdrawal. For people with more severe alcohol dependence, an in-patient withdrawal may be required [3,4]. After this, the treatment will be focused on reducing the risk of relapse, which can include psychosocial or pharmacological interventions and dealing with co-occurring issues that mediate alcohol consumption. These intervention options can include, but are not limited to, individual therapy, group sessions, community-based or residential rehabilitation programs, medications, network therapy, and promoting social support and the 12-step facilitation [5]. In addition to alcohol-specific treatment, individuals may also be diagnosed with other mental health disorders that will require treatment, for example, depression and anxiety [1,6-8]. The treatment options that can be delivered by each service providing specialist alcohol treatment will differ from service to service and will also be influenced by funding and resources [5]. An underlying factor of the alcohol treatment options available is the focus on reducing alcohol consumption and maintaining abstinence by increasing the individual's motivation to achieve this [9-11].

Common tools used by staff to establish a typical drinking pattern include timeline follow back (TLFB) [12] or breathalyzer measurements. However, these methods have limitations. The TLFB is limited in recall bias [13,14], and the breathalyzer is limited in the period it can cover, most likely only detecting if alcohol has been consumed in the past 24 hours or less before test administration. Outside of clinical services, another tool that can be used are other digital forms of alcohol management, such as smartphone apps. These have been identified with the potential to reduce alcohol consumption by tracking and providing feedback on the individuals’ behavior toward their goals [15]. However, they are limited by relying on self-report.

Now, transdermal alcohol sensors (TAS) are being developed and tested. These devices can measure alcohol consumption through sweat vapors on the skin and are similar in appearance to a health watch or tracker. TAS could be used to support alcohol treatment interventions, improve patient motivation, and reliably monitor alcohol consumption to provide an accurate and reliable regular data record for an extended period of time. TASs appear to have potential in clinical settings [16-20]. One brand (SCRAM [secure continuous remote alcohol monitoring]) has also been used effectively in the criminal justice system for alcohol-related offenses [21-25]. The SCRAM has been used in the criminal justice system in the United States since 2004 [24] and in England and Wales since 2020 [23].

Interest in TASs as a clinical tool is because they can address the limitations of current tools used to measure alcohol consumption. TAS can record alcohol consumption continuously as transdermal alcohol concentration (TAC) when worn appropriately, which could lead to weeks or months of detailed alcohol monitoring data. TASs have potential benefits and uses at different stages of treatment to provide detailed and accurate data for staff, tangible information to discuss with clients to consider drinking triggers and events, to monitor abstinence from alcohol during detoxification, as well as to enhance motivation for alcohol reduction or abstinence. However, there is currently no way of converting TAC to blood alcohol concentration (BAC) with the BACtrack Skyn [26].

A previous study our research team conducted, using TAS with alcohol-dependent individuals accessing treatment, suggests that patients find TAS acceptable to wear in their day-to-day lives without interference with their usual activities [27,28]. While that study had no aim to reduce alcohol consumption, in the interview, many patients stated that simply seeing the TAS on their wrist made them think about their alcohol consumption. Another study by Alessi et al [29] asked participants to complete a postwear survey after using SCRAM and found that 81% reported that the TAS helped them reduce drinking and that 75% would wear it for longer. The most common suggestions and concerns Alessi et al [29] found were related to the size and side effects of SCRAM. This was less commonly reported in our previous study, but the BACtrack Skyn is much smaller than SCRAM. Other studies looking at the acceptability and feasibility of the BACtrack Skyn support its use as an objective alcohol measurement tool [30] and for assessing alcohol use over an extended time frame (28 days) [31].

Another potential use of TAS in alcohol treatment is to use them as a method of delivering contingency management (CM). CM is an established treatment, recommended by the National Institute of Health and Care Excellence [32], and is effective for a range of substance use treatments [33-36]. However, although initially developed for use with alcohol use disorder (AUD) treatment, it has had limited adoption in routine clinical practice [37,38]. This is due to the nature of alcohol metabolism and its low detectability within the body. After alcohol consumption, the body rapidly metabolizes it, with the majority being eliminated by the liver. The other 2%-5% is eliminated through breath, urine, and sweat [39]. This means that alcohol is only detectable in the body for a short period. Currently used methods to detect alcohol in breath, blood, and urine have a relatively short time frame to detect alcohol. Thus, up until recently, to accurately implement CM in alcohol treatment, the individual would require frequent and multiple breath, blood, or urine tests daily to prove reduction or abstinence and correctly achieve CM rewards [36,38,40,41]. The implementation of this is not feasible with staff time and resources, and multiple daily visits would increase the burden on both patients and staff, as well as being potentially invasive. However, with the development of TAS, there is now the possibility to objectively monitor alcohol consumption 24/7 without these barriers [19,20,42-47].

Previous literature has started to explore TAS use to implement CM [19,20,42-47]. These studies found TAS successful in implementing the CM procedure and found that the CM intervention was able to significantly reduce alcohol consumption [19,20,43,44]. Of these studies, none involved alcohol-dependent participants. In total, 2 used recent drinking while intoxicated offenders with differing criteria on alcohol consumption, one AUDIT (alcohol use disorder identification test) score of 4+ [45] and the other AUDIT score of 8+ [42], 2 used human immunodeficiency virus-diagnosed individuals drinking higher levels of alcohol consumption [46,47], and the other 4 were classified as risky or heavy drinks with varying ways to define this [19,20,43,44]. The length of the TAS wear periods and CM length ranged from 1 to 4 months. Previous literature implementing CM with a TAS has used the SCRAM, which measures TAC at 30-minute intervals. The BACtrack Skyn measures every 20 seconds, allowing for a larger amount of data to be used when considering CM rewards and within the statistical analysis.

Including individuals with a current alcohol dependence diagnosis is important because this population may differ in several aspects from populations who drink alcohol but are not clinically dependent. Our study may provide insight into other considerations that laboratory or shorter duration studies do not. For example, participant compliance, motivation, and experience of wearing the TAS over a longer period. This study will explore the feasibility of using a TAS and providing CM to people attending community alcohol treatment services. The design was consistent with a previous study conducted by the same research team [27]. This previous study found that most participants were willing to wear the TAS for longer and that staff believe that to be used in alcohol treatment, it would be more clinically useful to have patients wear the TAS for longer than 1 week. Therefore, we have extended the wear time to 2 weeks in this study.

### Objectives and Hypothesis

The primary objective is to explore the feasibility, strengths, and limitations of using a TAS to monitor alcohol consumption in individuals in treatment for AUD with or without CM to promote low-risk consumption or abstinence.

The secondary objectives are to assess the acceptability of the TAS for individuals in treatment for AUD; to compare the accuracy of TAS compared to self-reported TLFB over a 2-week period in an alcohol-dependent clinical population; and to assess the implementation of CM to incentivize alcohol reduction or abstinence.

We will use a randomized controlled trial (RCT) trial design, randomizing participants into the CM or control group (1:1), to investigate this.

## Methods

### Trial Design

This is a RCT with a 1:1 allocation ratio to control and CM group.

### Participants, Eligibility Criteria, and Settings

The site participating in this study is South London and Maudsley (SLaM) NHS alcohol services, from June to December 2023. Specifically, 3 alcohol services: Wandsworth Community Drug and Alcohol Service, Pier Road Project, and the Alcohol Assertive Outreach Team from SLaM. Treatment staff in all services are specialists in the treatment of addiction behavior.

Individuals will be able to participate if they are attending one of the participating services and meet the following inclusion criteria: (1) receiving alcohol treatment for an alcohol use disorder in one of the participating South London alcohol services, (2) aged 18 years or older, (3) speaking English competently, (4) able to meet throughout the study period, (5) not currently participating in any other research trials, and (6) willing to provide informed consent to participate. Individuals will be excluded from participating if they meet any of the following exclusion criteria: (1) current use (past 4 weeks) of any illegal or addictive substances (excluding marijuana and tobacco and nicotine smoking), (2) under 18 years old, and (3) cannot speak English. This study focuses on adults in treatment for AUD; however, it was recognized and advised by staff consultation that if the exclusion criteria included all illegal drugs, including cannabis, this would reduce the number of potential participants within each service significantly. Therefore, it was decided to exclude the current use of any other illegal or addictive substance other than cannabis and tobacco and nicotine smoking.

### Ethical Considerations

This study was approved by the Cornwall and Plymouth research ethics committee (REC; reference 23/SW/0066). All participants provided written informed consent after reviewing consent documents with the research staff. Participants will be given a unique study ID that will be used to identify their data throughout the study. Only trained research staff will have access to the key that matches the participants’ ID. The key is password-protected and stored on a secure server at King’s College London. All participants will provide informed consent and will be aware that they can stop their participation at any point in the study without having to provide a reason to the researcher. All data will be password-protected and hardcopy files will be stored in a locked cabinet at King’s College London.

### Intervention

Both groups (control and CM groups) will wear the TAS for the same length of time, meeting with the researcher every other weekday. The TAS is not the intervention being measured but will be used to track participants’ alcohol use behavior to determine whether or not they have met the criteria for the CM rewards. The CM rewards are for abstinence or low drinking.

The CM group will be provided with vouchers if this target behavior occurs and is recorded by the TAS. The target behavior is very low drinking or abstinence, as measured by the TAS. The threshold for achieving the target behavior is below 115.660 TAC g/L (air). From here onwards, target behavior will be referred to as low or no drinking (defined as TAC below the set threshold of 115.660). This limit was chosen based on our previous research [48]. We decided to have the target behavior of low or no drinking as the typical amounts of alcohol being consumed by service users are much higher than this amount, and therefore, we considered a reduction to this amount still as an achievement for the service users.

For each day that the target behavior (low or no drinking) occurs, the CM group participants will be rewarded with a £5 (US $6.38) voucher. If target behavior occurs over consecutive days, they will be rewarded with bonus vouchers. If the target behavior occurs every single day for the study period, then they will be rewarded with another additional bonus voucher. In total, the participants could be provided with up to £180 (US $229.72) in CM rewards. The participant will not be eligible for the CM rewards if they remove the TAS for longer than 1 hour. The CM procedure is shown in Table 1.

In this example, day 1 is a Monday. If a participant was recruited on a Wednesday or Friday, the meetings would still occur on a similar schedule, always occurring on Mondays, Wednesdays, and Fridays only. This is to account for Skyn data storage.

All participants (control and CM groups) will additionally be compensated with a £5 (US $6.38) Love2Shop voucher at each meeting for their time and travel expenses reimbursed. All participants will also be given an additional £10 ($1=£0.79 GBP) Love2Shop voucher for returning the TAS at the end of participation to incentivize TAS return.

If the participant wishes to stop wearing the TAS, they will be told to contact the researcher and arrange a meeting to return the TAS. It will be clear that withdrawal will not interfere with their treatment or care at their service.

**Table 1:** Contingency management (CM) payment procedure plan (total: £180).

Day	Day of the week	CM for low or no drinking per day (£)	CM bonus for consecutive low or no drinking days (£5^a^ per day; £)	CM bonus for 14 days low or no drinking (£35 for 14 days; £)
1	Monday	5 (First meeting)	(First meeting)	—^b^
2	Tuesday	5	—	—
3	Wednesday	5 (Second meeting)	10 (Second meeting)	—
4	Thursday	5	—	—
5	Friday	5 (Third meeting)	10 (Third meeting)	—
6	Saturday	5	—	—
7	Sunday	5	—	—
8	Monday	5 (Fourth meeting)	15 (Fourth meeting)	—
9	Tuesday	5	—	—
10	Wednesday	5 (Fifth meeting)	10 (Fifth meeting)	—
11	Thursday	5	—	—
12	Friday	5 (Sixth meeting)	10 (Sixth meeting)	—
13	Saturday	5	—	—
14	Sunday	5	—	—
15	Monday	5 (Seventh meeting)	15 (Seventh meeting)	35 (Seventh meeting)
Sum	—	75	70	35

^a^£1=US $1.28.

^b^Not applicable.

### Procedure

**Figure 1:**
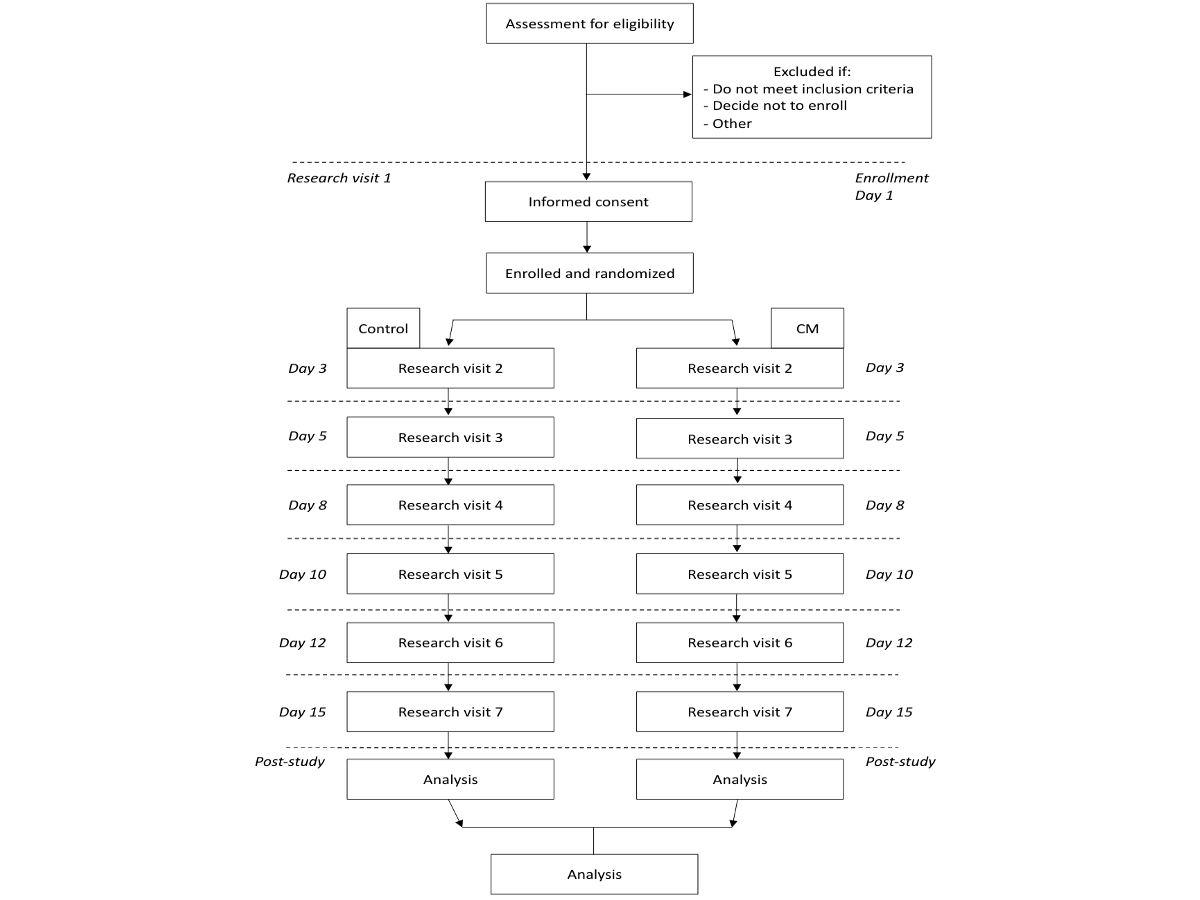
Flow chart of the study. Participants will be randomized into the control or CM group at the first research visit. The following research visits will occur every other weekday. In this example, the first research visit would occur on a Monday.

Figure 1 shows a flow chart of the study procedure. Participants will be recruited from the 3 SLaM alcohol services and approached by the researcher or service staff. Staff will be aware of the study and inclusion criteria and have participant information sheets to provide to individuals. The research team will then speak to the individual directly, answer any questions, and arrange a meeting if the individual meets the inclusion criteria and is willing to participate. The researcher will also attend group meetings at each service and be able to talk to service users directly if they are willing to be approached, describe the study, and provide participant information sheets.

**Figure 2:**
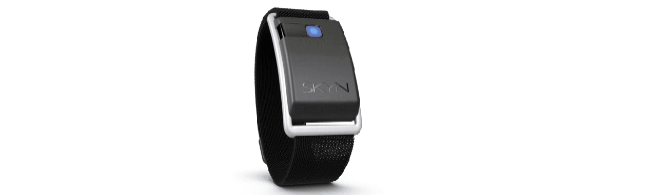
Image of the BACtrack Skyn (https://skyn.bactrack.com).

Randomization will take place at the first meeting after informed consent is provided. The participant will be enrolled as part of the control or CM group. Both groups will follow the same study procedure, conduct research visits, and wear the TAS. The difference is that the CM group could be provided with additional CM rewards when the target behavior occurs. At the first meeting, the participants will be trained on how to wear the TAS. The research visits occur every other weekday, a total of 7 times; for example, if starting on a Monday, they will meet Monday, Wednesday, Friday, Monday, Wednesday, Friday, and Monday. If the first meeting is on a Wednesday or Friday, the schedule will shift as needed, with meetings only occurring on Mondays, Wednesdays, and Fridays. At each meeting, the next meeting will be arranged, and a reminder text will be sent the day before. This is due to the storage capacity of the BACtrack Skyn TAS. It can store data for approximately 72 hours before data starts to be overwritten. Therefore, meetings must occur regularly to avoid data being overwritten. The TLFB will also be completed at each meeting (meetings 2-7) to record the past 2-3 days since the last meeting. An image of the BACtrack Skyn is shown in Figure 2. In the last meeting, the TAS will be returned, and a postwear survey will be completed. For the CM group, this will include questions on their experience with the CM rewards. The TAS recorded data will be analyzed by the research team and descriptive of missing data, removals (defined as skin temperature <30 degrees Celsius for longer than 2 minutes), and participant TAS adjustments.

### Outcomes

For the analysis of assessing the feasibility, strengths, and limitations of using a TAS to monitor alcohol consumption with or without CM to promote low-risk consumption of abstinence, both groups will have feasibility outcomes defined as follows: enrollment (recruitment rate, willingness of participants to enroll, willingness of clinicians to recruit participants); participation (enrolled participants who completed the intervention, attendance rate, response rate, compliance); tampering and malfunction rates (removal without replacement, tampering (turning it off), TAS malfunction, battery issues, number of times a participant had a query that required an extra contact or meeting with the researcher, and number of TAS returned); and the feasibility of using a TAS to measure CM target behavior and the acceptability of delivering CM to patients.

To assess acceptability, both groups will complete a postwear survey on wearing TAS, and those in the CM group will also complete a survey on their experience of receiving CM; these surveys are adapted from Alessi et al [29] and Miguel et al [49], respectively. Finally, to determine TAS accuracy, the TAC data recorded will be compared to the self-reported TLFB.

### Measures

#### BACtrack Skyn

The TAS that will be used is the BACtrack Skyn. It will be worn on the participant’s preferred wrist, but they will be allowed to change which wrist they wear it on during the study period. The Skyn will continuously measure the TAC while being worn, as well as skin temperature (Celsius). Output will be viewed at 1-minute intervals. The participants can remove the TAS at any time if they do not wish to wear it and will be required to remove it for bathing as it is not waterproof. The CM group will be told they can remove it once a day for up to 60 consecutive minutes to bathe and still be eligible for their CM reward. If removed for longer than an hour, then they will no longer be able to receive the CM reward, even if the other data suggest no alcohol consumption. If they wear the TAS according to this and the TAC does not increase above our set threshold of 115.660, they will be eligible for the CM reward for that day. We will explore various TAC criteria when defining a drinking event: TAS 15 (TAC>15, >15 minutes), TAS 60 (TAC>15, >60 minutes), and TAS 90 (TAC>15, >90 minutes).

To note, an alcohol drinking day defined by TAC is different from the CM intervention criteria, which allows for a low amount of drinking and has a higher TAC criterion. There are 2 distinctions: those in the CM group at each meeting will have their data examined for meeting the CM rewards criteria, and then in the analysis at the end after all data collection, all participants will have an accuracy analysis conducted where participant days will be defined as alcohol-drinking days (according to TAC/minute criteria) or nonalcohol drinking days.

#### TLFB

A TLFB will be completed at meetings 2-7 to assess self-reported alcohol consumption and compare it against the TAC data. The TLFB is a calendar-based measure to record self-reported substance use. Each date of the study period was completed at the following meeting. Any reported alcohol consumption on the TLFB will define that day as an alcohol-drinking day. Days will be recorded as 12 AM to 11:59 PM.

#### Postwear Surveys

Participants will complete a postwear survey on their experience of wearing the Skyn at their last meeting. This survey was adapted from Alessi et al [29]. If they were randomized to the CM group, they would also complete a survey on their CM experience. This study was adapted from Miguel et al [49].

#### Feasibility

Feasibility is defined by enrolment, participation, device tampering, removals, adjustments, malfunction rates, and the number of TAS returned (Textbox 1).

**Feasibility outcome definitions.**

**Enrolment**

- Recruitment rate, willingness of participants to enroll, and willingness of clinicians or services to recruit participants.

**Participation**

- Enrolled participants who attended meetings or intervention, follow-up rate, response rate, compliance, reasons for incomplete participant data (meeting nonattendance, data overwritten, and technical fault).

**Tampering**

- Tampering with the transdermal alcohol sensor (TAS) to hide alcohol consumption or to stop its recording.

**Removals**

- Removal (more than 2 minutes where the temperature was <30 °C).

**Malfunction**

- Device error, missing data due to technical fault, charging issues, and syncing issues.

**TAS return**

- Number of TAS successfully returned intact.

### Sample Size

The target is to recruit 30 participants within 6 months. This sample size was influenced by budget and time constraints and the number of participants considered suitable for meeting and carrying out the study’s aims of feasibility, as recommended for pilot studies by Lancaster et al and Browne [50-53].

### Randomization

This is a nonblinded study. Participants will be individually randomized into either the control group or the CM group (1:1 ratio). The randomization will be remote and overseen by an independent statistician using a sealed envelope randomization technique. The statistician (an independent researcher not involved in the study) generated a random list with Stata, with each allocation on a piece of A4 paper printed and sealed in the appropriate envelope (numbered 1-30). When a participant is enrolled, the researcher will then open the corresponding envelope to the participant's study ID for group allocation. Therefore, no member of the research team is aware of the group allocation until a participant enrolls on their first research visit.

### Recruitment

To achieve adequate participant enrollment and reach the target sample size, we plan to have regular communication with each participating service. We will attend each service regularly to inform staff of the ongoing recruitment and attend service user groups to discuss the research with eligible individuals.

### Data Collection and Management

In this study, there are 3 sources of alcohol measurement data collection.

The first is the TAS (BACtrack Skyn model T15). The TAS will collect TAC at 20-second intervals and average the appropriate 3 measurements to provide data at 1-minute intervals. This will be checked by the researcher at each research visit. For the CM group, these data will determine if they meet the criteria for the additional CM rewards. It will be checked to see if any TAC is above the low or no drinking threshold as well as for any removals. If the TAS is removed for periods of longer than 1 hour, then the participant will not be eligible for the CM rewards.

The second is the self-reported TLFB [12]. This will be used in the analysis to compare TAS accuracy to the TLFB.

The third potential alcohol collection method will be a breathalyzer. This study uses the Lion Alcometer SD-400 (Lion) with a fuel cell sensor (standard version) with a measuring range of 0.02-2.0 mg/L BrAC, an operating range of –5 to +45 C, and is calibrated 1 week before recruitment starts. The breathalyzer will only be used if the TAS malfunctions or does not record any data. We do not expect the TAS to malfunction, but if the TAS, for any reason, does not record hours of TAC data, we do not want this to impair the participants’ chance of meeting the criteria for the CM vouchers. If the TAS has not recorded the data, we will first ask the participant for a TLFB and if they have consumed alcohol in the past 2 days. If they respond “Yes, I have consumed alcohol,” then it will be marked as an alcohol-drinking day. If they say “No, I have not consumed alcohol,” we will ask them to do a breathalyzer to confirm low BrAC data. If the BrAC is below the United Kingdom drink drive limit, they will still be able to meet the criteria for the CM vouchers.

The data from each participant will be entered and coded as it is collected and will start after the first participant is enrolled. Personal data will be regarded as strictly confidential. Any data leaving the site will identify participants by their initials and unique identification code only. The study will comply with the Data Protection Act, 1988. The data custodian for this study is King’s College London. This data will be collected by EB.

### Statistical Analysis

Baseline and demographic variables will have descriptive statistics reported. Outcome measures will be conducted by intervention groups (control and CM) and then compared between the groups.

We will report on the feasibility measures stated earlier. Descriptive data from the postwear survey will be reported, and statistical comparisons of the postwear survey responses between groups will be performed using 2-tailed *t* tests. In instances where the data are ordinal or the assumptions of the paired samples *t* test are not met, the nonparametric alternative to the paired samples *t* test will be used, the Mann-Whitney *U* test. Given the small sample size and nature of this pilot study, the primary purpose of these results will be to inform the potential barriers and limitations and challenges faced in implementing CM for future work.

To assess TAS accuracy, the TAC data will be compared against the TLFB to address the secondary outcomes of accuracy. The analysis will focus on the sensitivity, specificity, positive predictive value, and negative predictive value of TAC compared to TLFB as the gold standard. Sensitivity in detecting alcohol events and specificity in classifying an alcohol-drinking day versus a nonalcohol-drinking day will be assessed. Recorded drinking and abstinent days will be analyzed using Spearman Rank correlations comparing different alcohol-drinking day TAC criteria. All statistical analyses will be conducted using SPSS (version 28; IBM Corp).

### Adverse Event Protocol

A data monitoring committee was not required for this study because it was a small pilot study. A data monitoring committee was not required in the ethical approval process.

Some previous literature reports slight irritation from wearing the TAS in certain activities, which the participant will be made aware of. There are no other expected medical complications. In the previous study conducted with the same design (minus CM) over 1 week instead of 2, there were no serious adverse events (SAE) reported by participants. The TASs are low risk for medical complications. There were side effects reported by 6 participants, which included rash and irritation of the skin from wearing the TAS. All participants were made aware that if the TAS remained uncomfortable, they could remove it. All participants said that after a day, irritation reduced and no further action was needed. Any SAE occurring during the study will be reported. In the case of a SAE that is related to the study or unexpected, the chief investigator will email the REC using the nonclinical trial of an investigation medicinal product safety report to the REC form. This would be sent within 15 days of the chief investigator becoming aware of the event. The research team includes supervision from a medical doctor.

## Results

This study has been designed to explore the feasibility, strengths, and limitations of using a TAS to monitor alcohol consumption in individuals in treatment for AUD with or without CM to promote low-risk consumption or abstinence. Our findings will contribute to the growing TAS literature on TAS implementing CM, expanding the literature to include the investigation of the BACtrack Skyn used to deliver CM in South London alcohol services. We completed the trial in December 2023; we recruited 32 participants; data analysis is underway; and the results are expected to be published by December 2024.

## Discussion

We hypothesize that TAS-delivered CM will be feasible to deliver and will be well-liked by participants. We anticipate that the TAS will be acceptable to wear for a 2-week period, with little to no challenge or side effects experienced. Side effects could possibly be related to the strap irritation against the wrist. In addition, we predict that the TAS will be more accurate in recording alcohol drinking days compared to self-reported drinking days.

Previous literature has started to investigate the accuracy, feasibility, and acceptability of TAS use and how TAS could be used to implement CM [19,20,42-46,54]. While previous evidence supports TAS accuracy, feasibility, and CM implementation with populations that range from social to heavy drinkers as defined by AUDIT scores and the NIAAA [55], there is yet to be a TAS study conducted in specialist alcohol services, particularly in the United Kingdom. To our knowledge, this is the first trial to establish the feasibility of TAS implementing CM, with clinically diagnosed alcohol-dependent individuals accessing treatment in the United Kingdom. The previous literature with TAS and CM has used the SCRAM [19,20,42-46,54]. This study will be the first to use the BACtrack Skyn for this purpose. There are 2 main differences between using SCRAM and BACtrack to deliver CM. The first is that SCRAM has longer data storage and can be downloaded remotely using a home phone landline or modem, allowing these studies to have weekly or less frequent research meetings but still check the data daily to deliver CM. The second is that SCRAM has an established alcohol event detection system to alert the researcher if there has been alcohol consumption. The BACtrack does not have this, and we had to determine a threshold for low drinking to use as CM eligibility for this study. These 2 SCRAM features may make it more feasible and less time-consuming and resource-intensive to deliver CM. However, the SCRAM is far bigger, bulkier, less stylish, and worn on the ankle, with a similar appearance to a house arrest monitor. This makes the SCRAM less suitable for use within clinical settings than a wrist-worn TAS, such as the BACtrack Skyn. Therefore, it will be useful to see how Skyn compares to the SCRAM to deliver CM and if this TAS brand too is feasible for this purpose.

Previous work by this research team demonstrated the acceptability of the BACtrack Skyn over 1 week with adults accessing treatment for alcohol dependence and a high correlation between the TAS and self-report [27,28]. This present protocol follows a similar design but over a slightly longer time (2 weeks rather than 1) and with the addition of the CM component. Therefore, due to the promising results of previous studies, we predict a similar high recruitment, attendance, and compliance among participants.

Our results will be of high relevance due to the increase in interest in the TAS field. Most of the TAS literature is being conducted in the United States with individuals without an alcohol dependence diagnosis. Therefore, this study will be highly important in determining whether the successful data shown in other literature translates to this population. It addresses a gap in the literature. Specifically, this pilot feasibility RCT will offer initial insights into the use of a wrist-worn TAS to monitor alcohol consumption and deliver CM for low or no drinking with an AUD population who are accessing treatment. Earlier studies have shown that the SCRAM ankle-worn TAS was feasible to implement CM, but the key difference in the removability of the BACtrack needs to be investigated. TASs provide a potential option to address the current barriers to implementing CM for alcohol treatment. TAS also has the potential to help adults accessing alcohol treatment to monitor, motivate, and reduce their alcohol consumption and maintain rates of abstinence.

This study will aim to determine the feasibility of conducting this study on a larger scale and any facilitating or barrier factors that should be considered in a larger study. For patient care, if shown to be effective, it will be important to consider future implementation within alcohol services as an option for treatment, with the necessary further investigation.

### Strengths

This study aims to demonstrate the feasible use of BACtrack Skyn over 2 weeks with individuals currently diagnosed with alcohol dependence and receiving alcohol treatment. Participants will wear the TAS in their natural settings, unsupervised. While the objective of this study is to assess the feasibility of a larger trial, the data collected will be able to provide more evidence of how this population wears, uses, and experiences a TAS. The study findings collected indicate high meeting attendance and TAS return rates, and no participants needing additional training for using the TAS after the baseline. This study’s results suggest continued support for the use of TAS within the population.

### Limitations

Participants will only be recruited if they are willing to wear the TAS from the start of the study period, but if during the study they change their mind, they can remove or stop wearing it. This means that no participants will be recruited who are not willing to attempt wearing the TAS. While this is not a limitation in some considerations, as TAS would be a voluntary treatment option for service users if implemented in services, it does mean that the postwear survey findings may be skewed more positively. Only those who were interested and willing to wear the TAS have the chance to complete the postwear survey at the end of the 2 weeks. However, participants being willing to wear the TAS for the study does not necessarily mean they are positive about the technology or that they will have a good experience wearing it. Therefore, while we note that the study design does exclude those who are not initially willing to attempt wearing a TAS, this may reflect a truer view of service users who would try wearing the TAS as part of alcohol treatment if TAS were to be implemented in clinical settings.

This trial hopes to add clinically relevant information about the use of a wrist-worn TAS to deliver CM to adults accessing alcohol treatment and determine the feasibility of this design for a future, larger trial. Of importance, this trial will be the first to use BACtrack Skyn to deliver CM and to deliver CM with a TAS within South London alcohol services.

## Appendix

Multimedia Appendix 1 CONSORT-eHEALTH checklist (V 1.6.1).

## Abbreviations

AUD: alcohol use disorder
AUDIT: alcohol use disorder identification test
BAC: blood alcohol concentration
CM: contingency management
NHS: National Health Service
RCT: randomized controlled trial
REC: research ethics committee
SAE: serious adverse event
SCRAM: secure continuous remote alcohol monitoring
SLaM: South London and Maudsley
TAC: transdermal alcohol concentration
TAS: transdermal alcohol sensor
TLFB: timeline follow back

## Data Availability

The research team, EB, PD, SP, and CD, only have access to the final trial data set. The data sets generated and analyzed during this study are available from the corresponding author on reasonable request.
